# Neck-specific training with a cognitive behavioural approach compared with prescribed physical activity in patients with cervical radiculopathy: a protocol of a prospective randomised clinical trial

**DOI:** 10.1186/1471-2474-15-274

**Published:** 2014-08-12

**Authors:** Åsa Dedering, Marie Halvorsen, Joshua Cleland, Mikael Svensson, Anneli Peolsson

**Affiliations:** Department of Physical Therapy, Karolinska University Hospital, 171 76 Stockholm, Sweden; Department of Neurobiology, Care Sciences and Society, Division of Physiotherapy, Karolinska Institutet, Stockholm, Sweden; Franklin Pierce University, Denver, USA; Department of Department of Clinical NeuroscienceKarolinska Instiutet, Karolinska Institutet, Stockholm, Sweden; Department of Neurosurgery, Karolinska University Hospital, Stockholm, Sweden; Department of Medical and Health Sciences, Division of Physiotherapy, Faculty of Health Sciences, Linköping University, Stockholm, Sweden

**Keywords:** Cervical radiculopathy, Conservative interventions, Neck pain, Outcome, Physical activity, Randomisation, Study protocol

## Abstract

**Background:**

Patients with cervical radiculopathy often have neck- and arm pain, neurological changes, activity limitations and difficulties in returning to work. Most patients are not candidates for surgery but are often treated with different conservative approaches and may be sick-listed for long periods. The purpose of the current study is to compare the effectiveness of neck-specific training versus prescribed physical activity.

**Methods/Design:**

The current protocol is a two armed intervention randomised clinical trial comparing the outcomes of patients receiving neck specific training or prescribed physical activity. A total of 144 patients with cervical radiculopathy will be randomly allocated to either of the two interventions. The interventions will be delivered by experienced physiotherapists and last 14 weeks. The primary outcome variable is neck- and arm pain intensity measured with a Visual Analogue Scale accompanied with secondary outcome measures of impairments and subjective health measurements collected before intervention and at 3, 6, 12 and 24 months after base-line assessment.

**Discussion:**

We anticipate that the results of this study will provide evidence to support recommendations as to the effectiveness of conservative interventions for patients with cervical radiculopathy.

**Trial registration:**

ClinicalTrials.gov identifier: NCT01831271

## Background

Cervical radiculopathy is mainly the result of cervical disc herniation or spondylosis which results in nerve root inflammation, impingement or both. Cervical radiculopathy has an annual incidence rate of 83.2 per 100,000 in the general population [1]. Patients often present with neck pain with radicular distribution in one or both upper extremities often together with paraesthesia, weakness or reflex changes. Certain patients with cervical disc herniation and/or spondylosis may suffer from prolonged functional impairments due to chronic pain, associated activity limitations, long periods of sick leave and difficulties in returning to work [1, 2].

Patients with cervical disc herniation and/or spondylosis suffering from cervical pain and radiculopathy are often treated conservatively [3]. If the symptoms persist and the radiological findings match the clinical symptoms, the patient may be referred for surgery [4]. The scientific evidence for the long term effect of treatment with physiotherapy, drugs or surgery is weak [5–7] and the benefit of surgery compared to conservative treatment is unclear [8]. Consequently, randomised controlled trials using valid and reliable measures to compare surgery with conservative treatment and to evaluate different conservative interventions are needed [9]. Only two prospective randomised controlled trials comparing physiotherapy with surgery have been performed [10, 11]. In the study by Bednarik [11] only patients with a diagnosis of myelopathy were included. Neither of the two studies found a significant difference between treatments at long-term follow-up. Peolsson et al. [12] and Engquist et al. [13] investigated the additional effect of anterior cervical decompression and fusion beyond the effect of a structured physiotherapy program which consisted of neck specific exercises and a behavioural approach in physical function and self-rated outcomes, respectively in patients with radiculopathy due to cervical disc disease. With the exception of less neck pain in the group with surgery plus structured physiotherapy there were no additional benefit at the 2 year follow-up of surgery compared with physiotherapy alone with regard to active range of neck motion, neck muscle endurance, hand related function [12], arm pain, neck specific function rated on Neck Disability Index or general outcome on the Odom scale [13].

The studies evaluating physical treatment for patients with cervical radiculopathy are of various quality [10, 13–22]. The interventions as well as criteria for participation are often poorly described and the number of patients in the trials is small [23]. Additionally, the physical treatment often consists of various interventions and is typically also combined with drugs and other treatments which make it difficult to determine the effects of a single intervention. Four of the studies were prospective randomised controlled trials [10, 17, 19, 21]. In the study by Persson [10] all patients had indications for a surgical intervention and the physiotherapy strategies were pragmatic, involving a number of different treatments. The current conclusion is that randomised controlled trials using valid and reliable outcome measures to compare different conservative interventions is needed [9, 24]. In patients with neck pain of mechanical origin there is evidence that neck specific training may be the single most effective management strategy [25]. In patients with chronic pain, a cognitive approach seems to be effective [26]. However, neither of these approaches has been examined in a patient population with cervical radiculopathy. Patients with cervical radiculopathy often have long-lasting pain before referral to a specialist hence, it is important to test these approaches and to compare them with the general recommendation to stay physically active.

Today, in clinical practice, the waiting times for patients with cervical radiculopathy to be evaluated by a surgeon are long and most of the patients are not surgical candidates. Many patients are therefore sick-listed for long periods of time with various treatment approaches or without any treatment at all. It has been suggested that a structured physiotherapy program should precede surgical intervention [12, 13] but this has not yet been scientifically evaluated.

The purpose of this randomised clinical trial will be to compare the effects of a neck-specific exercise program including a cognitive behavioral approach with prescribed, self-mediated and progressive physical activity approach in patients with cervical radiculopathy. We will include outcome assessment before and after the interventions at 3 and 6 months and 1- and 2 years post base-line.

## Methods

### Design

In a prospective, randomised clinical trial with follow-up at 3 and 6 months, and 1- and 2 years 144 patients with cervical radiculopathy will be included. The study will follow the CONSORT guidelines [27] and has been registered in ClinicalTrials.gov identifier: NCT01831271. The study has been approved of by the Regional Board of Ethics in Stockholm (Dnr 2009/1756-31/4).

### Participant selection

Patients with cervical pain and radiculopathy referred to the Neurosurgery Clinic at the Karolinska University Hospital will be invited to participate. A total of 144 consecutively selected patients will be included. Before inclusion all patients will undergo a standardized physical examination by a physiotherapist which will include a medical history, pain, sensory changes, reflexes, range of motion and muscle strength tests. Inclusion criteria are: 1) Magnetic Resonance Imaging (MRI) verified cervical disc disease showing cervical nerve root compression and 2) neck- and/or arm pain verified with a neck extension test or a neurodynamic provocation test positive Spurling test [28]. Exclusion criteria are: previous fracture or subluxation of the cervical spine, malignity, spinal tumour, spinal infection, previous surgery in the cervical spine, co-morbidity such as disease or symptoms contraindicated to perform the treatment program or the measurements, known drug abuse, lack of familiarity with the Swedish language, diagnosed psychiatric disorder. At the first visit to the physiotherapy department patients who satisfy eligibility criteria will be included if they agree to participate and provide informed consent.

### Sample size

The sample size is based on data from the “Swedish neck study” [12, 13]. A sample size of 56 in each group will have 80% power to detect differences in means of 15 mm assuming that the common standard deviation is 28 mm using two group t-test with a 0.05 two-sided significance level. A drop-out rate of 20% was calculated for.

### Randomisation

**Figure 1:**
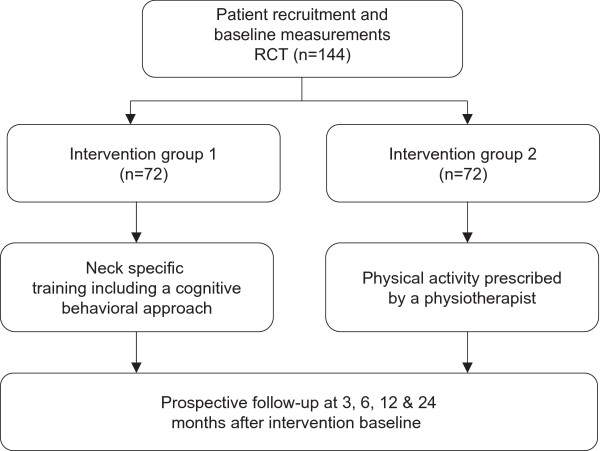
**Flow chart of the randomised clinical trial.**

After the baseline assessment, each patient will be randomised to one of two interventions: A) active physical rehabilitation with a neck specific exercise program including a cognitive behavioral approach (neck-specific training) or B) prescribed, self-mediated and progressive physical activity (prescribed physical activity). See flow chart (Figure 1). Randomisation will be done in blocks of eight according to a computer generated randomisation list prepared by an independent statistician not involved with subject recruitment. The sequence of allocation to either intervention will be concealed and performed by a person not involved in the testing or treatment of subjects in the project according to the random generated computer list and kept in numbered and sealed envelopes.

### Blinding

Group allocation will be concealed until the baseline measurement is completed. The patient will then receive a sealed envelope containing the group allocation. Blinding of the patients, the test leader and the treating physiotherapists participating in the study is not possible. First, because it will be obvious to the patient which the type of intervention they will receive and secondly, because the test leader also serves as contact person for the patients enrolled in the study and the patients could disclose their group assignment. To prevent bias from not blinding the two intervention arms would not be provided by the same physiotherapist or even the same geographical location i.e. prescribed physical activity would be given by physiotherapists other than those giving the intervention neck-specific training. The data transformation from paper questionnaires and physical examination protocols into data files will be handled by an independent person otherwise not involved in the study.

### Evaluation and test procedure

*Background data* that will be collected at the first visit of the study will include: gender, age, social situation, smoking habits, back pain, pain medication, pain history related to the neck problems, previous medical problems (differential-diagnosis to cervical disc disease), earlier treatments for the neck problems and its effects, work situation (such as type of work, workload due to the neck, work satisfaction and sick-leave), and physical activity/ exercise habits.

*Clinical measurements* that will be performed by an independent, blinded examiner before intervention, and at 3, 6, 12 and 24 months follow-up periods: Neurological examination, cervical range of motion [29], neck muscle endurance [30], hand strength [31] and balance [32, 33]. These measurements have good reliability and known reference data.

*Questionnaires* used have good reliability and validity (listed under secondary outcome measures). The questionnaires will be administered before intervention, 3, 6, 12 and 24 months after the start of the intervention.

### Primary outcome measure

The primary outcome measure is pain intensity. Intensity of neck pain and arm pain is measured on a Visual Analogue Scale (VAS) (0–100 mm) [34, 35]. Pain will be measured before intervention, 3, 6, 12 and 24 months after the start of the intervention.

### Secondary outcome measures

Additionally, the character of pain, number of pain locations and distribution of symptoms will be evaluated with a body diagram. Neck specific disability will be measured by the Neck Disability Index [36, 37]. Headache and dizziness will be measured on the VAS and Dizziness Handicap Inventory (DHI) [38]. Symptom satisfaction related to the neck problems will be rated on a seven-grade scale [39]. Self-efficacy will be measured on the Self-efficacy scale [40] and Exercise Self-efficacy scale. Fear-avoidance beliefs will be measured with the Fear Avoidance Beliefs questionnaire (FABQ) [41, 42] and Tampa Scale of Kinesiophobia (TSK) [42]. Hostility, anxiety and depression will be measured with the HAD scale [43, 44]. Coping strategies will be measured with the Coping Strategy Questionnaire (CSQ) [45]. Pain catastrophizing will be measured with the Pain Catastrophizing Scale (PCS) [46]. Health related quality of life assessed by the EuroQuol five dimensions self-classifier (EQ-5D) and current health state by the EuroQuol vertical VAS (0–100 mm) [47]. Physical activity will be measured with International Physical Activity Questionnaire (IPAQ short version) [48] and a scale for assessment of physical activity [49]. Patient specific goals will be measured with the Patient specific functional scale (PSFS) [50] and the Patient Goal Priority Questionnaire (PGPQ) [51]. Work ability will be measured by the Work Ability Index (WAI) [52].

*Additional questions* of fulfillment and satisfaction which will be asked after the intervention at the 6 and 24 months follow-ups such as: -adherence to the intervention, -treatment expectations fulfilled, - if they would recommend the intervention to patients with similar problems and if surgery or other interventions have been undertaken.

### Confidentiality

To guarantee the patients’ confidentiality all data collected will be coded and the codes will be locked in and stored separate from collected data.

### Intervention groups

Each physiotherapist who will treat patients in the study has been trained in the intervention program by the test leader who is a specialised physiotherapist at the specialist clinic. The entire treatment approach is documented in a manual of standard operating procedures and given to the treating physiotherapist. All exercise performed and progression of each will be registered in a diary. The written information is also available for the patients. For both groups the intervention period is 14 weeks.

#### Neck specific training

Active physical rehabilitation including neck-specific training with an additional cognitive behavioural approach will be included. The goal of the intervention is to improve physical functioning with specific respect to sensorimotor function, neck muscle strength and endurance as well as reducing pain. The active physiotherapy rehabilitation program consists of a standardised program (three times a week) with medical exercise therapy and if needed vestibular rehabilitation. At the start of the intervention motivational interviewing will be included. Additionally once a week during the first 14 weeks of the program (Week 1–14) the physiotherapist will educate the patient about physiology of pain, stress, exercise, breathing, relaxation, coping, pacing and ergonomics. The physiotherapist will also teach and have a discussion with the patient on how to manage the pain at home both physically with; heat, cold or TENS and psychologically with; relaxation training, exercises for increased body awareness as well as goal setting for better coping strategies and self-efficacy. Throughout the treatment program a cognitive approach from the physiotherapist according to theoretical behaviour change models will be used. Home exercises will also be prescribed. All physiotherapists will be provided with a well-defined frame of exercises with a standardised and structured progression. After a clinical examination the physiotherapist adjusts the program for each patient according to the selection of exercise and dosages chosen. From week 15–20 the patients have the opportunity to meet the physiotherapist for discussion, encouragement and help with exercise progression and otherwise do exercise on their own in the clinic or at home.

#### Prescribed physical activity

Physical activity will be formally prescribed by a physiotherapist and followed up according to customary routines [53, 54]. Prescribed physical activity is a tailored physical activity programme with the monitoring of progress and a follow-up. In guidelines for prescribing physical activity a cognitive behavioural approach (theoretical behaviour change model) with motivational interviewing is included. The interview includes exploratory talk, commitment/decision, life style change, health promotion, evaluation of readiness for change, reflection, assessment of motivation, patient specific goal assessment, conclusion and plan for follow-up at 14 weeks. Patients are guided by the physiotherapist to increase their overall activity and general strength with i.e. walking and other self-mediated activities and exercise. During the intervention and follow up period to 20 weeks the patients are free to contact their physiotherapist and the number of contacts will be registered.

The interventions will be led by experienced and specialised physiotherapists. The patients in the neck specific training group will have personal contacts with their physiotherapist whereas in the prescribed physical activity group they do not have scheduled regular contacts but the opportunity to contact the physiotherapist when needed. An earlier study of patients after lumbar disc surgery showed good results for an intervention based on home training when patients have received careful instructions and have access to the physiotherapist if questions arise [55].

### Data analysis

Outcome data from the two groups will be compared using an intention to treat analysis with all patients being analysed in the group to which they were originally randomly assigned even though they are lost to follow-up or non-compliance of the intervention or any other deviation from the protocol [56]. In the intention to treat analysis the technique multiple imputation of missing data will be used [57]. Continuous or discrete data will be compared with parametric or non-parametric statistical methods depending on possible skewness in the data distribution. Categorical data will be compared with non-parametric statistics. For relationships among several independent variables against a dependent variable multiple regression analysis will be used. Cost-effectiveness of the treatment models due to direct and indirect costs will be calculated.

## Discussion

The importance of the study for patients with cervical radiculopathy as well as society is significant as these individuals often experience severe, neuropathic pain resulting in both great personal and social costs. Many patients are sick-listed for long time periods.

The current study aims to supplement knowledge as to optimal treatment for patients with cervical radiculopathy. Treatment with surgery is also an identified knowledge gap in DUET (Record ID: 381431, published 2010-07-23) however not primarily addressed in the current study but could be effected secondarily as fewer patient might need surgery. The long-term effects of the study could potentially reduce the time of sick leave and improve the rate of patients returning to work and/or former activity by optimising treatments. Furthermore it may decrease the number of patients who will require surgery for their neck problems and thus reduce the mental, physical and societal costs.

The current trial is designed to minimise biases of risk in clinical trials by using randomisation, concealed allocation of patients, specified eligibility criteria and an intention to treat principle in analysis of the data. However, a complete blinding of the patients or the treating physiotherapists is not possible due to the types of intervention where it is obvious which kind of treatment one is receiving. Another potential limitation is that the study does not contain a true control or placebo group. The current patient group have pain and they are referred to the hospital due to their pain and significant findings on MRI. We potentially could have them on a waiting list for 14 weeks but that might result in increasing pain and disability. It would therefore not be ethical to assign some of them to a non-treatment group. Since there is minimal evidence and no consensus of how to best manage patients with cervical radiculopathy there exists considerable variation in what is currently being performed. The treatment differences which will occur in the current study are consistent with those sometimes used in clinical practice.

## Summary

This study uses a randomised clinical trial design to investigate the effectiveness of two types of conservative treatments for patients with cervical radiculopathy. The study will also investigate clinical features for possible prediction of a patient’s response to each treatment. The findings will enable evidence-based recommendations as to the effects of conservative interventions for patients with cervical radiculopathy. Furthermore, findings will provide direction for future research and treatment rationale for the patient population.
